# Mehrsegmentaler Zoster eines gesunden 20-Jährigen nach COVID-19-Impfung

**DOI:** 10.1007/s00105-022-04942-5

**Published:** 2022-01-14

**Authors:** V. Lebedeva, C. Müller, J. Dissemond

**Affiliations:** Klinik und Poliklinik für Dermatologie, Venerologie und Allergologie, Universitätsklinikum Essen, Hufelandstr. 55, 45122 Essen, Deutschland

In unserer Klinik stellte sich ein 20-jähriger Patient mit neu aufgetretenen schmerzhaften, vesikulösen Hautveränderungen zervikal linksseitig vor. In der Anamnese waren keine Vorerkrankungen bekannt; der Patient nahm keine Medikamente ein. Eine vermehrte UV-Exposition wurde verneint. Der Patient berichtete, 14 Tagen zuvor die erste SARS-CoV-2-Impfung mittels des Vektor-Impfstoffes Vaxzevria® (Fa. AstraZeneca, Cambridge, Großbritannien) erhalten zu haben.

## Klinischer Befund

**Figure 1:**
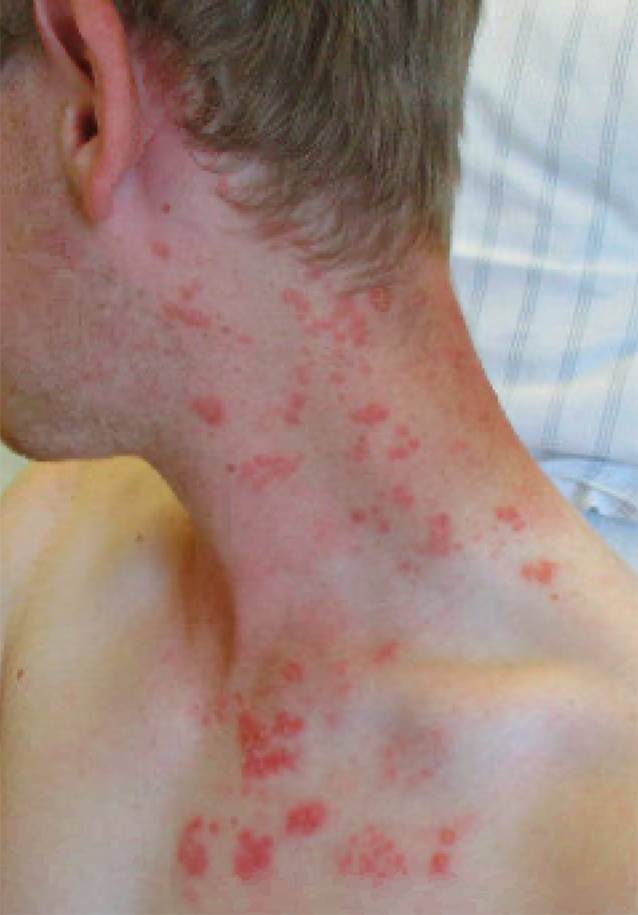

In der dermatologischen Untersuchung präsentierten sich im Bereich der Dermatome C3–4 linksseitig multiple, gruppiert stehende Vesikulae auf erythematösem Grund (Abb. 1). Laborchemisch fanden sich ebenso wie in der Röntgenuntersuchung des Thorax keine relevanten pathologischen Befunde. Serologisch wurde das Vorliegen einer Infektion mit humanem Immundefizienzvirus (HIV) sowie Hepatitis-Virus A–C und ausgeschlossen. Der mittels Polymerasekettenreaktion (PCR) auf eine COVID-19-Infektion untersuchte Nasen-Rachen-Abstrich war negativ.

## Diagnose und Therapie

Wir stellten die Diagnose eines mehrsegmentalen Zosters der Dermatome C3–4 linksseitig bei einem ansonsten gesunden 20-Jährigen nach SARS-CoV-2-Impfung und leiteten eine intravenöse Behandlung mittels Aciclovir 750 mg 3‑mal/Tag sowie eine begleitende analgetische Therapie ein, worunter sich der Befund rasch besserte.

## Diskussion

Ein Zoster wird durch die endogene Reaktivierung latenter Varizella-Zoster-Viren (VZV) bedingt, die nach einer Windpockenerkrankung oder -lebendimpfung zeitlebens in den Ganglien der Hinterwurzeln verbleiben. Zunehmendes Lebensalter, Immunsuppression und hohe UV-Exposition sind bekannte Provokationsfaktoren [1, 2]. Das Auftreten eines Zosters im Rahmen einer COVID-19-Infektion wurden bereits mehrfach beschrieben [3, 4]. Auch Vakzinierungen, wie beispielsweise gegen Tollwut, Influenza- und Hepatitis-A-Virus, können Trigger für Zoster darstellen. Als eine zentral relevante Ursache wurde eine transiente Lymphopenie nach Impfung diskutiert [5].

Seit Beginn der Vakzinierungen gegen SARS-CoV‑2, konnten wir in unserer Klinik wiederholt einen zeitlichen Zusammenhang mit dem Auftreten eines Zosters beobachten. In einer aktuellen Zusammenstellung der publizierten Literatur wurden 52 Patienten mit Zoster nach COVID-19-Impfung beschrieben. Lediglich einer der Patienten hatte eine Vakzinierung mittels Vektorimpfstoff erhalten, bei allen anderen Patienten waren es mRNA-Impfstoffe. Meist entwickelte sich der Zoster nach der ersten Impfung; der Zeitraum zwischen Vakzinierung und ersten Anzeichen des Zosters betrug 1 bis 26 Tage [6, 7].

Durch den Stufenplan der Ständigen Impfkommission (STIKO) am Robert Koch-Institut (RKI) wurde in Deutschland, ebenso wie in vielen Ländern weltweit, für die COVID-19-Impfstrategie eine Priorisierung insbesondere in Abhängigkeit von dem Lebensalter und den Vorerkrankungen festgelegt. Daher ist es nicht verwunderlich, dass die bislang publizierten Patienten mit Zoster nach Vakzinierung gegen SARS-CoV‑2 meist ein hohes Lebensalter und/oder entsprechende Vorerkrankungen aufwiesen. Es wurden jedoch zuletzt auch Fälle von Zoster nach COVID-19-Impfungen bei gesunden, jungen Patienten ohne Risikofaktoren publiziert. Die jüngsten dieser Patienten waren 21 bzw. 23 Jahre alt [5, 8]. Aktuell wurde in Deutschland die Priorisierung für die COVID-19-Vakzinierung aufgehoben, sodass sich nun jeder aus der Bevölkerung ab dem 12. Lebensjahr impfen lassen kann [9]. Es ist zu erwarten, dass in Zukunft auch noch jüngere Kinder geimpft werden.

Zu den häufigsten beschriebenen kutanen Nebenwirkungen nach COVID-19-Impfung zählen neben Erythemen (0,05 % Biontech vs. 0,06 % Moderna vs. 0,01 %) und Schwellungen (0,06 % Biontech vs. 0,1 % Moderna vs. 0,02 % AstraZeneca) auch makulopapulöse Arzneimittelexantheme (< 0,1 % Moderna) und Urtikaria (0,2 % Moderna) [10]. In einer aktuellen spanischen Studie konnten innerhalb eines Zeitraums von bis zu 21 Tagen 405 Patienten mit kutanen Nebenwirkungen identifiziert werden. Hier wurde eine VZV-Reaktivierung bei 10,1 % der Patienten beobachtet, wobei diese nach der Erst- häufiger als nach der Zweitimpfung auftrat (63,4 % vs. 36,6 %). In dieser Gruppe hatten 68,3 % der Betroffenen den Impfstoff von Biontech, 14,6 % von Moderna und 17,1 % von AstraZeneca erhalten [11].

Eine COVID-19-Infektion verläuft bei jüngeren Menschen in der Regel mild. Dennoch zeigte eine US-amerikanische Studie, dass 21 % der über 3200 Patienten mit einer COVID-19-Infektion im Alter zwischen 18 und 34 Jahren intensiv- und 10 % beatmungspflichtig waren. Verstorben sind rund 2,7 % der untersuchten jungen Patienten [12].

## Fazit für die Praxis

- Dieser Fallbericht zeigt den weltweit bisher jüngsten publizierten gesunden Patienten mit Zoster nach COVID-19-Impfung.
- Klinisch tätige Ärzte sollten wissen, dass es auch bei jungen, ansonsten gesunden Menschen nach COVID-19-Impfung zu einem Zoster als seltene Komplikation kommen kann.
- Das Zoster-Risiko sollte den Patienten und ggf. Eltern vor einer anstehenden Impfung bekannt sein und bei der Entscheidung mit berücksichtigt werden.
